# Association between environmental contaminants and health outcomes in indigenous populations of the Circumpolar North

**DOI:** 10.3402/ijch.v73.25808

**Published:** 2014-12-05

**Authors:** Kavita Singh, Peter Bjerregaard, Hing Man Chan

**Affiliations:** 1 Department of Biology, University of Ottawa, Ottawa, ON, Canada; 2 Department of Health, Centre for Health Research in Greenland, Greenland Government and University of Greenland, Nuuk, Greenland; 3 Canada Research Chair in Toxicology and Environmental Health, Center for Advanced Research in Environmental Genomics, University of Ottawa, Ottawa, ON, Canada

**Keywords:** epidemiology, review, environment, human health, polychlorinated biphenyls, pesticides

## Abstract

**Background:**

Since the 1990s, research has been carried out to monitor environmental contaminants and their effects on human health in the Arctic. Although evidence shows that Arctic indigenous peoples are exposed to higher levels of contaminants and do worse on several dimensions of health compared with other populations, the contribution of such exposures on adverse outcomes is unclear.

**Objective:**

The purpose of this review is to provide a synopsis of the published epidemiological literature that has examined association between environmental contaminants and health outcomes in Arctic indigenous populations.

**Design:**

A literature search was conducted in OVID Medline (1946-January 2014) using search terms that combined concepts of contaminant and indigenous populations in the Arctic. No language or date restrictions were applied. The reference lists of review articles were hand-searched.

**Results:**

Of 559 citations, 60 studies were relevant. The studies fell under the following categories: paediatric (n=18), reproductive health (n=18), obstetrics and gynaecology (n=9), cardiology (n=7), bone health (n=2), oncology (n=2), endocrinology (n=2) and other (n=2). All studies, except one from Arctic Finland, were either from Nunavik or Greenland. Most studies assessed polychlorinated biphenyls (n=43) and organochlorine pesticides (n=29). Fewer studies examined heavy metals, perfluorinated compounds, or polybrominated diphenyl ethers. Details of study results for each health category are provided.

**Conclusions:**

It is difficult to make conclusive statements about the effects of environmental contaminants on health due to mixed results, small number of studies and studies being restricted to a small number of regions. Meta-analytical synthesis of the evidence should be considered for priority contaminants and health outcomes. The following research gaps should be addressed in future studies: association of contaminants and health in other Arctic regions (i.e. Inuvialuit Settlement Region, Nunavut, Nunatsiavut, Alaska, European North and Russian North); assessment of contaminants on chronic diseases; inclusion of clinical endpoints in assessments; and assessment of the emerging contaminants of perfluorinated compounds and polybrominated diphenyl ethers.

Indigenous populations residing in the Arctic regions (e.g. the Kalaallit in Greenland, the Inuit and Inuvialuit in Canada, the Inupiat and Yupik in Alaska, and the Yuit in Siberia) are encountering a myriad of environmental and health-related challenges, previously unknown to these communities. Despite residing in locations distant from industrial activity, Arctic populations are especially vulnerable to environmental contaminant exposure. Contaminants undergo long-range transport from warmer to colder regions, thus making the Arctic a sink for contaminant deposition. Also, lipophilic chemicals, such as persistent organic pollutants, collect in fatty tissues of animals and bioaccumulate at the higher ends of the food chain. The consumption of marine mammals by Arctic populations thus leads to direct contaminant exposure. The presence of contaminants in the North has garnered the attention of researchers. Since the 1990s, multidisciplinary international projects have been implemented to monitor contaminants and potential impacts on human health in the Arctic (1). The most important initiative, the Arctic Monitoring Assessment Programme (AMAP), includes all Arctic countries and had led to a series of reports detailing pollution and climate change issues (2–4).

Studies have shown that indigenous populations in the Arctic have higher body burden of contaminants than other populations. Donaldson et al. reported higher concentrations of heavy metals and persistent organic pollutants in Inuit mothers from Inuvik compared with Dene/Metis and non-aboriginal women (5). High levels were also measured in Inuit mothers from Nunavik and the Baffin region of Nunavut, although levels decreased from 1992 to 2007 (5). From the Adult Inuit Health Survey, Laird et al. reported several-fold higher contaminant levels in Canadian Inuit compared with the general Canadian population (6). In the Russian North, contaminant levels were measured in blood, cord blood, and breast milk of indigenous women, and blood of adult indigenous populations and controls residing in urban locations (3). Mothers from the Chukotsky District had the highest contaminant concentrations in blood and breast milk. Overall, blood contaminant levels in indigenous populations of Arctic Russia were similar to those of coastal Greenland and Northern Canada (3).

Many of the health issues faced by Arctic populations are related to the shift from traditional to modern lifestyles, which result in more sedentary behaviours and consumption of higher-fat content, nutrient-poor market foods. The shift in lifestyle has resulted in a corresponding shift from infectious diseases to chronic, non-communicable diseases such as diabetes, cardiovascular disease and cancer (3,7–9). The prevalence of diabetes among the Inuit in Canada, for instance, has historically been below the national average, but has now increased to an equivalent level (10). Chronic disease risk factors, such as obesity and smoking, are prevalent among Northern communities. In 2009–2010, 58.3% of Inuit adults 18 years of age or older in Canada were overweight or obese, compared with 51.9% among the non-aboriginal population (10). Furthermore, 59.6% of Inuit adults reported being physically inactive during leisure time (49.7% among non-aboriginal populations), 78.4% ate less than the recommended number of servings of fruits and vegetables per day, and 44.4% smoked tobacco (16% among non-aboriginal populations) (10).

While it is clear that Arctic populations are exposed to higher levels of environmental contaminants and fare worse on certain dimensions of health than other populations, the resulting impact of these exposures on health are still ambiguous. Contaminants do not exist in isolation – they are but one additional risk factor among a plethora of other factors, such as diet, smoking, genetic predisposition and socioeconomic status, that contribute to disease. Isolating the effects of contaminants from these other contributors can be difficult in epidemiological studies of small populations (5). Nonetheless, the ongoing inquiry into the adverse health effects of contaminants is vital so that research methods undergo refinement to detect small, but relevant, associations and scientifically based messages of exposure and strategies for mitigation are conveyed to the populations at risk.

Because the Arctic indigenous peoples have distinct cultural practices, diet and socioeconomic factors that make them vulnerable to contaminant exposure and adverse health outcomes, the purpose of this review is to provide a synopsis of the published epidemiological research, available to date, that has examined association between environmental contaminants and health outcomes in these populations. The primary aim is to identify health topics that have received greater research attention and areas where more research is needed. Donaldson et al. provided an overview of the health effects of contaminants, though not restricted to Arctic populations (5). This review updates this previous work. In addition, this review provides a systematic assessment of the literature in Arctic populations and graphical representations to show research gaps.

## Methods

A comprehensive search of the published literature was conducted in OVID Medline (1946-January 2014) using search terms that combined the concepts of contaminant and indigenous Arctic populations. To describe Arctic indigenous populations, we included the following search terms: Inuit, Dene, Kalaallit, Yupik, Buryat, Chukchi, Evenk, Khanty, Koriak, Koryak, Nenet, Sami, Yukaghir, Mansi, Nganasan and similar terms with variations of spelling. No language or date restrictions were applied. The titles and abstracts of all citations yielded by the searches were reviewed. Primary studies that examined the effect of at least one environmental contaminant on any type of health outcome in humans were retained and included in the review. The reference lists of selected review articles were also hand-searched for relevant primary studies. Studies reporting body burden, intake of contaminants, or health outcomes only were not included in this review.

The studies were organized into broad health categories. In addition, studies were tabulated according to location and environmental contaminant. Basic data characteristics, such as study design, sample size, contaminant exposure level and overall results, were extracted for individual studies and presented in tabular format.

Major cohort studies on the health effects of polychlorinated biphenyls (PCBs) and mercury have been conducted in the Faroe Islands (11–13). Based on cultural and geographical differences, they are not included in this review, which focuses on indigenous populations of the circumpolar Arctic countries. In addition, several studies on the health effects of contaminants in Norway and Russia (14–18) have been conducted, but they have not been included in this review because they were not limited to the indigenous populations of those regions.

## Results

The Medline search yielded a total of 559 citations. Of these, and the references hand-searched from review articles, 60 primary studies were deemed to be relevant for this review (i.e. these studies included an evaluation of at least one association between an environmental contaminant and a health outcome in an indigenous population in the Arctic). Other excluded studies primarily measured levels of environmental contaminants in humans but did not link such measurements with health outcomes. The studies fell under the following broad categories: paediatric, reproductive health, obstetrics and gynaecology, cardiology, bone health, oncology, endocrinology and other. Studies that examined health outcomes of environmental contaminants in children under the age of 18 years of age were classified under the paediatric category and omitted from the other categories. Similarly, studies that examined health outcomes in pregnant women were classified under the obstetrics and gynaecology category and omitted from other categories (e.g. assessment of thyroid function in pregnant women was classified under obstetrics and gynaecology rather than endocrinology).

**Fig. 1:**
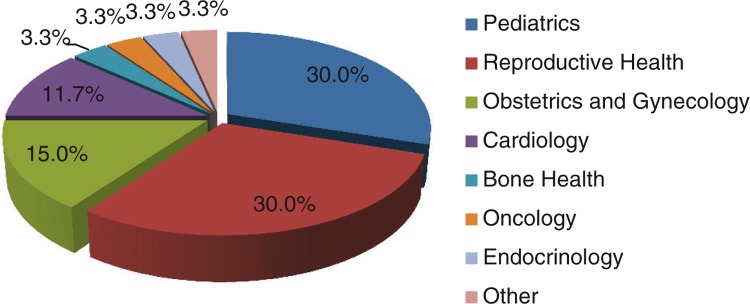
Studies by health category.

Figure 1 depicts the number of studies falling under each health category. Most studies examined associations between contaminants and health outcomes in paediatrics (N=18) and on reproductive health outcomes (N=18). Subsequent categories were obstetrics and gynaecology (N=9) and cardiology (N=7). Only 2 studies were found in each of bone heath, oncology and endocrinology. In addition, 2 studies that examined markers of oxidative stress were classified in the other category.

**Fig. 2:**
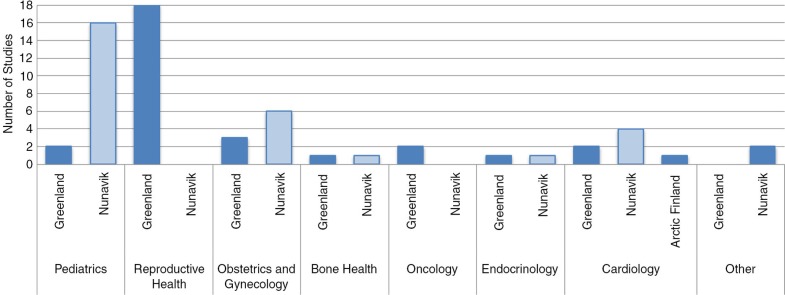
Studies by health category and location.

All studies were from either Nunavik or Greenland, except for one study from Arctic Finland (Fig. 2). The majority of paediatric studies examined Inuit from Nunavik whereas all reproductive health studies examined Inuit from Greenland.

**Fig. 3:**
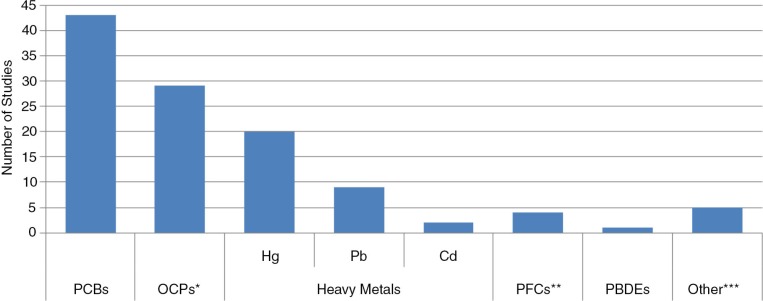
Studies by contaminant. *OCPs – e.g. p,p′-DDE, DDT, HCB, PCP. **PFCs – e.g. PFOA, PFOS. ***Other – octachlorostyrene, dioxin-like compounds, POPs (specific compounds unspecified). Cd=cadmium; DDE=dichlorodiphenyldichloroethylene; DDT=dichlorodiphenyltrichloroethane; HCB=hexachlorobenzene; Hg=mercury; OCPs=organochlorine pesticides; Pb=lead; PBDEs=polybrominated diphenyl ethers; PCBs=polychlorinated biphenyls; PCP=pentachlorophenol; PFCs=perfluorinated compounds; PFOA=perfluorooctanoic acid; PFOS=perfluorooctanesulfonate; POPs=persistent organic pollutant.

As shown in Fig. 3, most studies examined the adverse health impacts of PCBs (N=43) and organochlorine pesticides (N=29). Smaller number of studies examined heavy metals (Hg, N=20; Pb; N=9; Cd, N=2), perfluorinated compounds (N=4), polybrominated diphenyl ethers (PBDEs) (N=1) and other contaminants including octachlorostyrene, dioxin-like compounds and unspecified persistent organic pollutants (N=5). Aside from cardiology, PCBs were examined by the largest number of studies in all health categories. Organochlorine pesticides or heavy metals fared second.

### Paediatric

Paediatric studies have examined associations between PCBs or organochlorine pesticides with infections and immune status (19–22), neurological function (23–26), indicators of behaviour (27–29) and thyroid function (30) (Table I). Studies have also looked at heavy metals (i.e. Hg or Pb) and their effects on blood pressure and heart rate variability (31), neurological function (23,24,25,26,32,33,34) and behaviour (27,29,35,36). Most studies were prospective cohorts of mother–child pairs that followed children from birth to infants or childhood. Sample sizes ranged from 10 to 400. Contaminant levels were measured in maternal blood, cord blood, breast milk and/or child blood and mercury hair levels were measured in some studies. Most analyses adjusted for several demographic (e.g. age, gender), lifestyle (e.g. smoking) and other contaminant exposures.

**Table I:** Paediatric studies

Study	Contaminant	Outcome	Population	Results*
Infections				
Jensen (19)	PCBs and OCPs	Otitis media	Mother–child pairs (n=400) and children 4–10 years of age at follow-up (n=223) *(participation rate: 56%)*	No association
Dallaire (20)	PCBs	Acute respiratory infection	Children 0–5 years of age (n=343) *(participation rate: 70%)*	Cord PCB-153 Acute otitis media: RR=1.37, 95% CI: 1.20–1.55 LRTI: RR=1.44, 95% CI: 1.20–1.72 URTI or hospitalization: No association
Dallaire (21)	PCBs and p,p′-DDE	Acute infections	Infants during the first 12 months of life (n=199)	Maternal PCB-153 LRTI (first 6 months): RR=1.68, 95% CI: 1.00–2.81 (3rd exposure quartile) GI infection (12 months): RR=1.59, 95% CI: 1.01–2.49 (3rd exposure quartile) Maternal p,p′-DDE URTI (first 6 months): RR=1.56, 95% CI: 1.05–2.33 (2nd exposure quartile) URTI (12 months): RR=1.34, 95% CI: 1.00–1.78 (2nd exposure quartile) Otitis media (first 6 months): RR=1.83, 95% CI: 1.09–3.07 (3rd exposure quartile) All infections (first 6 months): RR=1.33, 95% CI: 1.03–1.73 (3rd exposure quartile) GI infection (12 months): RR=1.59, 95% CI: 1.03–2.47 (2nd exposure quartile) Child PCB or p,p′-DDE: No association
Dewailly (22)	PCBs and OCPs	Infections and immune status	Newborns followed up to 12 months of age (n=171)	Breast milk Mirex Acute otitis media (4–7 months): RR=1.88, 95% CI: 1.14–3.08 (2nd exposure tertile) Breast milk dieldrin Acute otitis media (4–7 months): RR=1.75, 95% CI: 1.05–2.91 (3rd exposure tertile) Breast milk HCB Acute otitis media (first 12 months): RR=1.49, 95% CI: 1.10–2.03) (3rd exposure tertile) Breast milk p,p′-DDE Acute otitis media (first 12 months): RR=1.52, 95% CI: 1.05–2.22) (3rd exposure tertile) Breast milk PCBs: No association Bronchopulmonary diseases: No association Immunological parameters: No association
Behavioural				
Boucher (27)	PCBs, Hg and Pb	ADHD	Children followed from birth to 11 years of age (n=279) *(participation rate: 95%)*	Cord Hg Attention problems: Positive association (β=0.13, 95% CI: 0.00–0.25) Disruptive Behaviour Disorders score: OR=2.87, 95% CI: 1.04–7.94 (ADHD-inattentive type, 3rd exposure tertile) OR=2.92, 95% CI: 1.07–8.04 (ADHD-hyperactive impulsive type, 3rd exposure tertile) Child blood Pb Externalizing problems: Positive association (β=0.14, 95% CI: 0.01–0.26) Disruptive Behaviour Disorders score: OR=5.52, 95% CI: 1.38–22.12 (ADHD-hyperactive impulsive type, 3rd exposure tertile) PCB-153: No association
Verner (28)	PCBs	Attention and activity	Infants followed from birth to 11 months of age (n=168)	Cord PCB-153 Inattention: Positive association (Spearman’s correlation=0.205) Infant blood PCB-153 Non-elicited activity: Positive association (Spearman’s correlation=0.182 at 11 months of age)
Plusquellec (29)	PCBs, Hg and Pb	Behavioural indicators	Children followed from birth to 5 years of age (n=110)	Cord PCB-153 Happiness: Inverse association (β=−0.22) Anxiety: Positive association (β=0.26) Global activity latency: Inverse association (β=−0.25) Positive affect rate: Inverse association (β=−0.24) Child blood Pb Impulsivity: Positive association (β=0.20) Irritability: Positive association (β=0.20) Inattention: Positive association (β=0.21) Hg: No association
Plusquellec (35)	Pb	Behavioural function	Infants 11 months of age (n=169)	Cord Pb Frenetic activity: Associated with greater activity (β=−0.16) Off-task duration: Positive association (β=0.17) Off-task latency: Inverse association (β=−0.20) No association with other measures
Fraser (36)	Pb	Motor function and behaviour	Children 5 years of age (n=110)	Child blood Pb Impulsivity: Positive association (Pearson correlation=0.25) Activity: Positive association (Pearson correlation=0.25) No association with attention level
Neurological				
Ethier (23)	PCBs, Hg and Pb	Visual brain development	Children followed from birth to 10–13 years of age (n=172)	Cord Hg N75 amplitude at 95% contract level: Positive association (β=0.206) N75 latency at 12% contrast level: Positive association (β=0.285) Cord Pb N150 latency: Positive association (β=0.238, 0.209, 0.251 at 95%, 12% and 4% contrast levels respectively) PCB-153: No association
Boucher (24)	PCBs	Response inhibition error monitoring	Children followed from birth to a mean age of 11 years (n=196)	Child plasma PCB-153 Reaction times: Positive association (i.e. slower times) (β=0.18 for go trials) Amplitudes of P_e_ and P_c_ response-related potentials: Inverse association (β=−0.16 and −0.20 respectively) Cord Pb: Correct responses: Inverse association (i.e. fewer correct responses) (β=−0.21 and −0.17 for correct go and no-go responses respectively) Child blood Pb Correct responses on no-go trials (false alarms): Inverse association (more false alarms) (β=−0.16) P3 amplitudes: Inverse association (β=−0.16 and −0.23 for go and no-go trials respectively Hg: No association
Boucher (25)	PCBs and Hg	Information processing	Children followed from birth to 11 years of age (n=118)	Cord PCB: No association with sample as a whole P3b amplitude in subgroup of children breast-fed for >3 months: Inverse association (β=−0.32) Cord Hg Reaction times: Positive association (i.e. slower time) (β=0.15) False alarms: Inverse association (i.e. fewer false alarms) (β=−0.21) N1 latency: Positive association (β=0.29) N1 amplitude: Inverse association (β=−032)
Boucher (32)	Pb	Working memory	Children 5 years of age (n=104) or 11 years of age (n=201) *(participation rate: 26% for ERP at 5 years and 55% for ERP at 11 years)*	Cord Pb (5 years) P3b amplitude: Inverse association (β=−0.38) Child blood Pb (5 years) P3b latency: Positive association (β=0.37) Pb (11 years): No association
Saint-Amour (33)	PCBs and Hg	Visual brain processing	Children followed from birth to 5–6 years of age (n=102)	Child plasma PCB-153 P100 latency at 95% contrast: Positive association (β=2.50) N150 latency at 12% contrast: Positive association (β=5.58) N75–P100 amplitude at 95% contrast: Inverse association (β=−3.74) Cord Hg P100 latency at 30% contrast: Positive association (β=3.34) Child blood Hg N75 early latency at 95% and 30% contrasts: Inverse association (β=−3.90 and −3.18, respectively) P100 latency at 95% and 30% contrasts: Inverse association (β=−3.26 and −3.94, respectively)
Després (26)	PCBs, Hg and Pb	Neuromotor functions	Children followed from birth to pre-school (n=110)	Child plasma PCB-153 Sway oscillations: Positive association (β=0.22 for transversal sway) Child blood Hg Tremor: Positive association (β=0.20) Child blood Pb Reaction time: Positive association (i.e. slower time) (β=0.24) Sway oscillations: Positive association (β=0.24, 0.22, 0.26 for velocity, sagittal and transversal sway) Movement irregularity: Positive association (β=0.22) Coherence between hands: Inverse association (β=−0.29) Synkinesis: Positive association (β=0.23) Tremor: Positive association (β=0.24)
Weihe (34)	Hg	Neurobehavioural performance	Children followed from birth to 7–12 years of age (n=21) and children 7–12 years of age (n=22)	Maternal hair Hg Hand–eye coordination (error score): Positive association (r=0.44) Peak latencies on brainstem auditory evoked potentials prolonged at higher exposure levels when data combined with other cohorts (Faroes and Madeira)
Cardiovascular and endocrine				
Valera (31)	Hg	Blood pressure and heart rate variability	Children followed from birth to 11 years of age (n=226) *(participation rate: 46%)*	Child blood Hg HRV – low frequency: Inverse association (β=−0.24) HRV – SDNN: Inverse association (β=−0.28) HRV – SDANN: Inverse association (β=−0.32) HRV – CVRR: Inverse association (β=−0.06) Blood Pressure: No association
Sandau (30)	PCBs, PCP and octachlorostyrene	Thyroid function	Newborns (n=10)	Cord PCP T_3_, TBG and fT_4_: Inverse association (r=−0.55, −0.44, −0.51 respectively) Cord ΣPCBs and ΣPCB hydroxylated metabolites TSH: Inverse association (r=−0.46 and −0.45 respectively) Sum of all cord chlorinated phenolic compounds T_3_ and fT_4_: Inverse association (r=−0.48 and −0.47 respectively)

ADHD=attention deficit hyperactivity disorder; CI=confidence interval; CVRR=coefficient of variation of R-R intervals; DDE=dichlorodiphenyldichloroethylene; ERP=event-related potential; fT_4_=free thyroxine; GI=gastrointestinal; HCB=hexachlorobenzene; Hg=mercury; HRV=heart rate variability; LRTI=lower respiratory tract infection; OCPs=organochlorine pesticides; OR=odds ratio; Pb=lead; PCBs=polychlorinated biphenyls; PCP=pentachlorophenol; RR=relative risk; SDANN=standard deviation of R-R intervals measured over 5 min periods; SDNN=standard deviation of R-R intervals; T_3_=triiodothyronine; TBG=thyroxine-binding globulin; TSH=thyroid-stimulating hormone; URTI=upper respiratory tract infection

* Adjusted estimates are presented where available. Presented estimates are statistically significant at p≤0.05 level.

#### Infections and immune system

For infection-related outcomes studies presented mixed results. One study found no association between PCBs and organochlorine pesticides with otitis media (19). However, Dallaire et al. found that cord PCB-153 was associated with higher incidence of acute otitis media (adjusted RR=1.37, 95% CI: 1.20–1.55 for most exposed compared with least exposed) and lower respiratory tract infections (LRTI) (adjusted RR=1.44, 95% CI: 1.20–1.72) (20). In addition, maternal PCB-153 was positively associated with LRTI in the first 6 months of life (adjusted RR=1.68, 95% CI: 1.00–2.81, for 3rd exposure quartile compared with least exposed) and gastrointestinal (GI) infections at 12 months (adjusted RR=1.59, 95% CI: 1.01–2.49, for 3rd exposure quartile compared with least exposed) (21). In the same study, maternal p,p′-dichlorodiphenyldichloroethylene (p,p′-DDE) was found to be associated positively with upper respiratory tract infections (URTI), otitis media, and all infections during the first 6 months of life and URTI and GI infection at 12 months (21). Dewailly et al. found that organochlorine pesticides were associated with increased risk of acute otitis media but not with bronchopulmonary diseases (22).

#### Behaviour

Boucher et al. observed a higher incidence of attention deficit hyperactivity disorder (ADHD) in children 11 years of age exposed to mercury prenatally and among those with higher levels of blood lead (27). No association was seen between ADHD and PCB-153 (27). However, other studies found that PCB-153 was associated positively with inattention and non-elicited activity in infants aged 11 months (28) and with unhappiness and anxiety in children aged 5 years (29). Child blood lead levels have been observed to be associated with greater impulsivity (29), irritability (29) and activity (36). Plusquellec et al. also found a positive association with inattention (29), but this was not detected in other studies (27,36). Prenatal exposure to lead was associated with greater frenetic activity and off-task duration in infants (35).

#### Neurological

No association was detected between PCB-153 and visual brain development, but cord mercury and lead levels were associated with certain visual evoked potentials (i.e. N75 amplitude, N75 latency and N150 latency) (23). Saint-Amour et al. found that child plasma PCB-153 and cord and child blood mercury levels were associated with alterations in visual brain processing, as assessed by visual evoked potentials (33). Child plasma levels of PCB-153 have also been significantly associated with slower reaction times (24) and an increase in transversal sway oscillations (26). Lead levels in cord and child blood have been associated with fewer correct responses on go and no-go trials (24) and adverse neuromotor functions (26). Mercury has also been found to adversely affect information processing (e.g. slower reaction times, incorrect responses) (25), increase tremors (26) and hand–eye coordination error score (34).

#### Cardiovascular and endocrine

Only one study assessed the effect of mercury on cardiovascular outcomes in children who were followed from birth to 11 years of age (31). No association was found between mercury levels and blood pressure. However, child mercury level was associated with certain measures of heart rate variability. A small study in newborns (n=10) evaluated the effect of PCB, pentachlorophenol (PCP) and octachlorostyrene exposure on thyroid function (30). PCP, ΣPCBs and ΣPCB hydroxylated metabolites were found to be inversely associated with thyroid hormones.

### Reproductive health

Studies of reproductive health have primarily examined the effects of PCBs and organochlorine pesticides on oestrogen, androgen or aryl hydrocarbon receptor activities (37–42), fertility and markers of male reproductive function (43,44), sperm deoxyribonucleic acid (DNA) damage and apoptotic markers (45,46), epididymal and accessory sex gland functions (47), reproductive hormone levels (48) and sperm Y:X ratio (49) (Table II). Sample sizes range from 37 to 598. Several studies analyzed a cohort of Greenlandic Inuit men from the INUENDO project, which also recruited cohorts of men from Warsaw, Kharkiv and Swedish fishermen for comparisons. The results presented in Table II are for the Inuit cohort only.

**Table II:** Studies of reproductive health

Study	Contaminant	Outcome	Population	Results*
Mocevic (53)	Hg	Semen quality and reproduc. hormones	Male partners of pregnant women (n=194)	Inhibin B: Positive association (β=0.074, 95% CI: 0.021–0.126) No association with other semen characteristics or reproductive hormones
Kvist (54)	PFOA and PFOS	Sperm Y:X ratio	Male partners of pregnant women (n=201) *(participation rate: 78.5%)*	PFOS Inverse association (i.e. lower Y:X ratio) (β=−0.002, 95% CI: −0.004–0.000) PFOA: No association
Krüger (37)	PCBs and OCPs	ER, AR and AhR function	Men and women (n=247) *(participation rate: 41% of all Greenlandic AMAP population and 74% for XAR and XAR*_*comp*_*outcomes)*	ΣPCBs ER transactivity (males): Inverse association (β=−0.36 and −0.24 for XER and XER_comp_ respectively) ER transactivity (females): Inverse association (β=−0.41 for XER_comp_) AhR transactivity (males): No association AhR transactivity (females): Inverse association (β=−0.61) ΣOCPs ER transactivity (males): Inverse association (β=−0.34 for XER) ER transactivity (females): Inverse association (β=−0.36 for XER_comp_) AhR transactivity (males): Inverse association (β=−0.29) AhR transactivity (females): Inverse association (β=−0.55) AR transactivity: No association
Krüger (38)	PCBs and OCPs	ER and AR transactivity	Men and women (n=240) *(participation rate: 82% for XAR/XER outcome)*	ΣPCBs ER transactivity (males and females): No association upon adjustment AR transactivity (males): Inverse association (β=−0.42) AR transactivity (females): No association ΣOCPs ER transactivity (males): No association ER transactivity (females): Inverse association when adjusted for age (β=−0.24) AR transactivity (males): Inverse association (β=−0.36) AR transactivity (females): No association
Bonde (43)	PCBs and p,p′-DDE	Fertility and markers of male reproductive function	Pregnant women and their spouses (n=598) *(participation rate: 90%)*	PCB-153 LH: Positive association (β=0.07, 95% CI: 0.02–0.12) Sperm volume: Inverse association (β=−0.11, 95% CI: −0.2 to −0.04) Progressive sperm: Inverse association (β=−4, 95% CI: −6 to −1) Sperm counts: Low counts in subgroup of men with short androgen receptor CAG repeat length Neutral α-glucosidase activity in seminal plasma: Inverse association (β=−0.1, 95% CI: −0.2 to −0.0) No association with other measures (e.g. time to conceive, apoptotic markers, sperm chromatin integrity)
				p,p′-DDE Inhibin B: Positive association (β=6.4, 95% CI: 1.7–13.8) Free testosterone: Positive association (β=0.02, 95% CI: 0.0–0.04) Sperm volume: Inverse association (β=−0.04, 95% CI: −0.16 to −0.01) Progressive sperm: Inverse association (β=−0.01, 95% CI: unclear to −0.6) No association with other measures (e.g. sperm chromatin integrity, apoptotic markers, epididymal and accessory sex gland function)
Krüger (50)	POPs (evaluated as effects on ER, AR and AhR)	Sperm chromatin integrity	Male spouses of pregnant women (n=53)	DNA fragmentation index: Inverse association with ER and AhR activities DNA stainability: No association
Long (39)	PCBs and OCPs	AhR transactivity	Men and women 18–77 years of age (n=357) *(participation rate: 48% for AhR*_*comp*_*outcome)*	ΣPCBs: No association (males or females) with AhR-TEQ Inverse association with AhR_comp_ in males and females combined (β=−0.18) ΣOCPs Inverse association with AhR-TEQ (β=−0.31) in males; No association in females Inverse association with AhR-TEQ (β=−0.21) in males and females combined Inverse association with AhR_comp_ in males and females combined (β=−0.18)
Krüger (40)	PCBs and p,p′-DDE	Serum xenoandrogenic activity	Male spouses of pregnant women (n=37)	PCB-153: No association p,p′-DDE: No association
Toft (52)	POPs (evaluated as effects on ER, AR and AhR)	Semen quality	Male spouses of pregnant women (n=54)	No association specifically in Inuit. When data combined across all 4 populations (Warsaw, Greenland, Kharkiv, Sweden), ER activity associated with increase in sperm concentration and motility.
Long (51)	POPs (evaluated as effects on ER, AR and AhR)	Sperm DNA damage and Sperm apoptotic markers	Male spouses of pregnant women (n=54)	DNA damage: Inverse association with ER and AhR Bcl-xL marker: Inverse association with AR (Spearman’s correlation=−0.46) No association with the sperm apoptotic marker, Fas
Long (41)	PCBs and p,p′-DDE	AhR activity	Males (n=75)	PCB-153: No association p,p′-DDE: No association
Stronati (45)	PCBs and p,p′-DDE	Sperm DNA fragmentation and sperm apoptotic markers	Male spouses of pregnant women (n=200) *(participation rate: 79%)*	PCB-153: No association p,p′-DDE: No association
Elzanaty (47)	PCBs and p,p′-DDE	Epididymal function and Accessory sex gland function	Male spouses of pregnant women (n=163)	PCB-153 Epididymal marker (neutral-α glucosidase): Inverse association (β=−0.2, 95% CI: −0.3 to −0.04) No association with PSA, zinc, or fructose p,p′-DDE: No association
Giwercman (48)	PCBs and p,p′-DDE	Reproductive hormone levels	Male spouses of pregnant women (n=258) *(participation rate: 79%)*	PCB-153 LH: Positive association for highest exposure group compared with lowest exposure group (MD=1.4 IU/L, 95% CI: 1.1–1.7 IU/L) No association with other reproductive hormones p,p′-DDE Free testosterone: Positive association (β=0.011, 95% CI: 0.004–0.024) Inhibin B: Positive association for highest exposure group compared with lowest exposure group (MD=35 ng/L, 95% CI: 1.5–69 ng/L) No association with other reproductive hormones
Bonefeld-Jorgensen (42)	PCBs and p,p′-DDE	Serum xenoestrogenic activity	Male spouses of pregnant women (n=72)	PCB-153: No association p,p′-DDE: Inverse association (Spearman’s correlation=−0.29 for XER)
Tiido (49)	PCBs and p,p′-DDE	Sperm Y:X ratio	Male spouses of pregnant women (n=157)	PCB-153: No association p,p′-DDE: No association
Toft (44)	PCBs and p,p′-DDE	Fertility	Pregnant women (n=598) and their spouses (n=201) *(participation rate: 87%)*	PCB-153: No association p,p′-DDE: No association
Spanò (46)	PCBs and p,p′-DDE	Sperm chromatin integrity	Male spouses of pregnant women (n=193)	PCB-153: No association p,p′-DDE: No association

AhR=aryl hydrocarbon receptor; AMAP=Arctic Monitoring Assessment Programme; AR=androgen receptor; CI=confidence interval; DDE=dichlorodiphenyldichloroethylene; DNA=deoxyribonucleic acid; ER=oestrogen receptor; Hg=mercury; IU=international unit; LH=luteinizing hormone; MD=mean difference; OCPs=organochlorine pesticides; PCBs=polychlorinated biphenyls; PFOA=perfluorooctanoic acid; PFOS=perfluorooctanesulfonate; POPs=persistent organic pollutants; PSA=prostate specific antigen.

* Adjusted estimates are presented where available. Presented estimates are statistically significant at p≤0.05 level.

The effects of PCB-153 on reproductive outcomes have been mixed in the published literature. Some studies have found statistically significant inverse associations with sperm volume, progressive sperm and neutral α-glucosidase activity in seminal plasma (an epididymal marker) (43,47) and statistically significant positive associations with luteinizing hormone (43,48). However, no associations have been found with time to conceive, apoptotic markers, sperm chromatin integrity, other reproductive hormones, sperm Y:X ratio, or fertility (43–49). Studies also present mixed results for p,p′-DDE. Significant positive associations were present for inhibin B and free testosterone, and inverse associations for sperm volume and progressive sperm (43,48). No associations were found for sperm chromatin integrity, apoptotic markers, epididymal and accessory sex gland function, sperm DNA fragmentation, other reproductive hormones, sperm Y:X ratio, or fertility (43–49).

Unspecified persistent organic pollutants, as evaluated by effects on oestrogen, androgen, or aryl hydrocarbon receptors, were inversely associated with DNA fragmentation index, DNA damage and the anti-apoptotic marker, Bcl-xL (50,51). No associations were found for DNA stainability, semen quality, or the apoptotic marker, Fas (50–52).

Additional studies examined the effects of mercury on semen quality and reproductive hormones (53) and perfluorinated compounds on sperm Y:X ratio (54). Mercury was positively associated with inhibin B but not with other semen characteristics or reproductive hormones (53). Perfluorooctanesulfonate (PFOS) was associated with lower sperm Y:X ratio, though no association was present for perfluorooctanoic acid (PFOA) (54).

### Obstetrics and gynaecology

Table III provides details of studies that examined environmental contaminants and effects on obstetrical or gynaecological outcomes. A total of 9 studies, with sample sizes ranging from 22 to 572, were found (55–63). Several inverse associations were observed between contaminants and birth outcomes. PCBs, organochlorine pesticides and mercury were associated with shorter duration of pregnancy and foetal growth (55,60,63). In addition, PCB-153 and p,p′-DDE were associated with lower birth weight and shorter gestational age, but not with risk of preterm birth, in women with live singleton deliveries (56).

**Table III:** Studies of obstetrics and gynaecology

Study	Contaminant	Outcome	Population	Results*
Dallaire (55)	PCBs, HCB and Hg	Foetal Growth and pregnancy duration	Pregnant women (n = 248) *(participation rate: 59%)*	Cord PCB-153, HCB and Hg Duration of pregnancy: Inverse association (β = − 0.17 to −0.20) Fatal growth (mediated through shorter gestation period): Inverse association for length (β = − 0.16 to −0.18 for PCB-153 and HCB respectively)
Wojtyniak (56)	PCBs and p,p′-DDE	Birth weight, gestational age and preterm birth	Women with singleton live births (n = 572) *(participation rate: 86%)*	PCB-153 Birth weight: Inverse association (β = − 59.2, 95% CI: −100.6 to −17.8) Gestational age: Inverse association (β = − 0.2, 95% CI: −0.4–0.0) p,p′-DDE Birth weight: Inverse association (β = − 39.4, 95% CI: −79.0–0.2) Gestational age: Inverse association (β = − 0.2, 95% CI: −0.4–0.0) No association with preterm birth
Dallaire (57)	PCBs, HCB and PCP	Thyroid hormone levels	Pregnant women (n = 107) and infants up to 7 months of age (n = 130)	Thyroid hormone levels in women at delivery: Maternal PCB hydroxylated metabolites T_3_: Positive association (β = 0.57) No association with other thyroid hormones Maternal PCB-153, HCB and PCP: No association Thyroid hormone levels in umbilical cord: Maternal and cord PCB-153 TBG: Inverse association (β = − 0.25 for maternal PCB-153 and −0.26 for cord PCB-153) No association with other thyroid hormones Maternal PCP fT_4_: Inverse association (β = − 0.59) No association with other thyroid hormones Maternal or cord HCB and PCB hydroxylated metabolites/cord PCP: No association Infant PCB-153 or HCB: No association
Toft (58)	PCBs and p,p′-DDE	Menstrual cycle	Pregnant women presenting for antenatal care at local hospitals (n = 454) *(participation rate: 90%)*	PCB-153 Long menstrual cycles: Inverse association (OR = 0.7, 95% CI: 0.5–0.96) No association with average cycle length, irregular cycles, or short cycles p,p′-DDE Long menstrual cycles: Inverse association (OR = 0.7, 95% CI: 0.5–0.99) No association with average cycle length, irregular cycles, or short cycles
Lucas (59)	PCBs and Hg	Birth weight and gestational age	Pregnant women (n = 491) *(participation rate: 30% for gestational age outcome)*	Cord PCB-153 Birth weight: Positive association in unadjusted analysis Hg: No association
Muckle (60) (Abstract only)	PCBs, OCPs and Hg	Developmental effects	Not provided	OCs Physical growth at birth: Inverse association (estimate not provided) Duration of pregnancy: Inverse association (estimate not provided) Hg: No information provided
Pereg (61)	PCBs	Placental CYP1A1 activity	Pregnant women admitted to hospital upon delivery (n = 35)	No association
Lagueux (62)	PCBs and OCPs	Placental CYP1A1 activity and DNA adducts	Women giving birth in regional hospitals (n = 22)	PCB-153, p,p′-DDE, HCB CYP1A1 activity: Positive association in moderate smokers (R^2^=0.21, 0.51, 0.38 respectively) PCB-118 CYP1A1 activity: Positive association in heavy smokers (R^2^=0.30) Associated with bulky DNA adduct formation in non-smokers and moderate smokers across all cohorts, including non-Inuit p,p′-DDE Associated with less bulky DNA adducts across all cohorts, including non-Inuit
Foldspang (63)	Hg	Gestational length and birth weight	Mothers with singleton deliveries (n = 376) *(participation rate: 45.9% newborns represented from one district)*	Birth weight: Inverse association (β = − 7.1 for maternal blood mercury and −4.2 for offspring blood mercury) No association with gestational length

CI=confidence interval; DDE=dichlorodiphenyldichloroethylene; DNA=deoxyribonucleic acid; fT_4_=free thyroxine; HCB=hexachlorobenzene; Hg=mercury; OCs=organochlorines; OCPs=organochlorine pesticides; OR=odds ratio; PCBs=polychlorinated biphenyls; PCP=pentachlorophenol; T_3_=triiodothyronine; TBG=thyroxine-binding globulin.

* Adjusted estimates are presented where available. Presented estimates are statistically significant at p ≤ 0.05 level.

Thyroid hormone levels in relation to maternal, cord, or infant levels of PCBs and organochlorine pesticides were evaluated in one study (57). Maternal PCB hydroxylated metabolites, PCB-153, and PCP and cord PCB-153 were associated with thyroid hormones. However, no associations were found with infant contaminant levels.

Menstrual cycle characteristics of women presenting for antenatal care at hospitals were examined by Toft et al. (58). PCB-153 and p,p′-DDE were associated with fewer long menstrual cycles. No association was found between PCB-153 or p,p′-DDE with average cycle length, irregular cycles, or short cycles.

Two small studies (n=35 and n=22) measured placental CYP1A1 activity (61,62). One study reported no association with PCBs (61), whereas the other study found increased CYP1A1 activity with higher exposures to PCB-153/PCB-118 and organochlorine pesticides, depending on smoking status (62).

An AMAP report of persistent toxic substances in indigenous populations of the Russian North is available (3). This report was not formally included in this review as it was not in the published literature domain, but results from the report are summarized here. Premature births were associated with blood lead levels >3.0 µg/L, cadmium >1.0 µg/L and Aroclor 1,260 >5.0 µg/L. Reduced birth weight was associated with cadmium and Aroclor at the same thresholds. In addition, women with stillbirths or births with serious structural malformations had PCB, dichlorodiphenyltrichloroethane (DDT) and mercury levels 1.7–2.0 times higher compared with women with no adverse birth outcomes. An examination of dose-response relationship found that sum of PCBs in maternal serum >2.0 µg/L was associated with birth weight and gestational age and >4.0 µg/L with fatal pregnancy outcomes. In addition, the ArcRisk project, which examined environmental contaminants in Eastern Arctic regions, conducted meta-analyses of the association between PCBs and sex ratio and birth weight (4). No association was found between PCBs and sex ratio, but maternal PCB concentration was associated with low birth weight.

### Cardiology

Surrogate outcomes of cardiovascular disease, most commonly blood pressure, have been assessed in several studies (64–69) (Table IV). Other outcomes include resting heart rate and pulse pressure, plasma lipids and cardiac autonomic activity (65,68,70). The studies were large, with sample sizes ranging from 230 to 1,861.

**Table IV:** Studies of cardiology, endocrinology, bone health, oncology and oxidative stress in adults

Study	Contaminant	Outcome	Poulation	Results*
Cardiology				
Valera (64)	PCBs and OCPs	Hypertension (≥140/90 mm Hg or taking anti-hypertensive medication)	Men and women ≥18 years of age (n=1,614) *(participation rate: 52% from larger population)*	ΣDL-PCBs Hypertension: Positive association in youngest age category (18–39 years) (OR=1.34, 95% CI: 1.03–1.74) Σnon-DL-PCBs Hypertension: Inverse association in oldest age category (≥40 years) (OR=0.81, 95% CI: 0.66–0.99) DDT Hypertension: Positive association in youngest age category (18–39 years) (OR=1.42, 95% CI: 1.08–1.85) Aldrin, α-chlordane, γ-chlordane Hypertension: Inverse associations in youngest age category (18–39 years) (OR=0.39, 95% CI: 0.20–0.78; OR=0.38, 95% CI: 0.19–0.75; OR=0.10, 95% CI: 0.03–0.38 respectively) Mirex Hypertension: Inverse association in oldest age category (≥40 years) (OR=0.80, 95% CI: 0.69–0.93) No association across all age categories
Valera (65)	Hg	Blood pressure, resting heart rate and pulse pressure	Men and women ≥18 years of age (n=313) *(participation rate: 41%)*	Resting heart rate: Positive association Diastolic blood pressure: Inverse association No association with systolic blood pressure or pulse pressure
Nielsen (66)	Hg	Blood pressure	Men and women 30–69 years of age (n=1,861) *(participation rate: 67.5%)*	Diastolic blood pressure: Inverse association in men (β=−0.04) Hypertension: Inverse association in men (OR=0.99, 95% CI: 0.98–0.99) No association in women
Valera (67)	Hg	Blood pressure	Men and women ≥18 years of age (n=732) *(participation rate: 55%)*	Systolic blood pressure: Positive association (β=2.14, 95% CI: 0.94–3.33) Diastolic blood pressure: No association
Château-Degat (70)	PFOS	Plasma lipids	Men and women 18–74 years of age (n=723) *(participation rate: 68%)*	Triacylglycerol: Inverse association in women (β=−0.0014) HDL-C: Positive association (β=0.0042 and 0.0016 for women and men respectively) Ratio TC/HDL-C: Inverse association (β=−0.0035) No association with LDL-C or non-HDL-C
Valera (68)	Hg	Blood pressure and cardiac autonomic activity	Men and women ≥40 years of age (n=280) *(participation rate: 59%)*	SDANN: Inverse association (β=−0.086, 95% CI: −0.16 to −0.01) Systolic blood pressure: Positive association (β=4.77, 95% CI: 1.12–8.42) Pulse pressure: Positive association (β=3.40, 95% CI: 1.11–5.69) No association with diastolic blood pressure or other measures of cardiac autonomic activity
Luoma (69)	Cd	Blood pressure and arterial hypertensive disease	Reindeer herders 20–82 years of age in Arctic Finland (n=230) *(participation rate: 14%)*	Systolic blood pressure: Positive association Diastolic blood pressure: No association
Endocrinology				
Jørgensen (71)	PCBs and OCPs	Glucose intolerance	Men and women (mean age 49 years) (n=692) *(participation rate: 67%)*	PCBs and OCPs 2-hour insulin: Inverse association DL-PCBs and non-DL-PCBs HOMA-B: Inverse association. No association with mean fasting glucose, mean 2-hour glucose, or mean fasting insulin. No association with IGT or diabetes.
Dallaire (72)	PCBs, OCPs, PBDEs, PFOS and dioxin-like compounds	Thyroid function	Men and women ≥18 years of age (n=623) *(participation rate: 50%)*	PCBs T_3_: Inverse association (β=−0.020) (inverse association also for PCB metabolites) TBG: Inverse association (β=−0.037) (inverse association also for PCB metabolites) HCB T_3_: Inverse association (β=−0.030) fT_4_: Inverse association (β=−0.017) TBG: Inverse association (β=−0.054) β-HCH T_3_: Inverse association (β=−0.028) TBG: Inverse association (β=−0.051) PFOS T_3_: Inverse association (β=−0.017) fT_4_: Positive association (β=0.014) TBG: Inverse association (β=−0.034) TSH: Inverse association (β=−0.102) No associations with p,p′-DDE or PBDEs after full adjustment for confounders
Bone health				
Paunescu (73)	DL-PCBs (evaluated as effects on AhR)	Bone strength	Women 35–72 years of age (n=194)	No association
Côté (74)	PCBs and OCPs	Bone ultrasound	Peri- and post-menopausal women 49–64 years of age (n=153)	PCB-156 Broadband ultrasound attenuation (indication of bone density and architecture): Inverse association (β=−8.12) Speed of sound (indication of bone density and elasticity): Inverse association (β=−22.68) Stiffness index (indication of rigidity of bone structure): Inverse association (β=−11.95) PCB-153: No association
Oncology				
Bonefeld-Jorgensen (75)	PCBs, OCPs, PFCs and heavy metals	Breast cancer	Cases (n=31) and controls (n=115)	PCBs Highest quartile of exposure significantly higher for cases than controls. Otherwise no association.
				PFCs Positive association with breast cancer (OR=1.03, 95% CI: 1.001–1.07 for PFOS and 1.03, 95% CI: 1.00–1.05 for Σperfluorosulfonated acids) ΣPOPs Positive association (OR=1.02, 95% CI: 1.01–1.04) OCPs: No association Heavy metals: No association
Rusiecki (76)	PCBs and OCPs	DNA methylation (*Alu* and *LINE-1* assays)	Mostly males 19–67 years of age (n=70)	ΣPCBs Inverse association on *Alu* assay (β=−0.56) (i.e. DNA hypomethylation) p,p′-DDE, DDT, other pesticides Inverse associations on *Alu* assay (β=−0.26 to −0.75) ΣPOPs Inverse association on *Alu* assay (β=−0.48) No association on *LINE-1* assays
Other				
Bélanger (78)	PCBs and Hg	Oxidative stress (redox status of CoQ10 and vitamin E)	Men and women (majority women) (n=99)	ΣPCBs Total tocopherols: Positive association (β=2.68) α-Tocopherol: Positive association (β=4.12) Ratio α-tocopheryl quinone/α-tocopherol: Inverse association (β=−0.41) Overall, no evidence of oxidative stress Hg α-Tocopheryl quinone: Inverse association (β=−0.30) Overall, no evidence of oxidative stress No association with ubiquinol-10 or ubiquinone-10 and PCBs or Hg
Bélanger (77)	PCBs and Hg	Oxidative stress (plasma oxidized LDL-C, homocysteine, glutathione peroxidase, glutathione reductase and glutathione)	Men and women (majority women) (n=99)	PCBs Oxidized LDL-C: Positive association (β=0.11) Hg: No association

AhR=aryl hydrocarbon receptor; Cd=cadmium; CI=confidence interval; DDE=dichlorodiphenyldichloroethylene; DDT=dichlorodiphenyltrichloroethane; DL-PCBs=dioxin-like polychlorinated biphenyls; DNA=deoxyribonucleic acid; fT_4_=free thyroxine; HCB=hexachlorobenzene; HCH=hexachlorocyclohexane; HDL-C=high density lipoprotein cholesterol; Hg=mercury; HOMA-B=homeostasis model assessment of beta cell function; IGT=impaired glucose tolerance; LDL-C=low density lipoprotein cholesterol; OCPs=organochlorine pesticides; OR=odds ratio; PBDEs=polybrominated diphenyl ethers; PCBs=polychlorinated biphenyls; PFCs=perfluorinated compounds; PFOS=perfluorooctanesulfonate; POPs=persistent organic pollutants; SDANN=standard deviation of the average RR intervals calculated over 5 minute periods; T_3_=triiodothyronine; TBG=thyroxine-binding globulin; TC=total cholesterol; TSH=thyroid-stimulating hormone.

* Adjusted estimates are presented where available. Presented estimates are statistically significant at p≤0.05 level.

Two studies, including the largest one with 1,861 participants, found that mercury was associated with lower diastolic blood pressure (65,66). However, 2 other studies (n=732 and n=280) found no association with diastolic blood pressure (67,68). Results were similarly inconclusive for systolic blood pressure (i.e. positive association in 2 studies but no association in others) (65–68). A study of reindeer herders in northernmost Arctic Finland found that hypertensive subjects had higher blood cadmium levels compared with normotensives and that blood cadmium was positively associated with systolic blood pressure when adjusted for age, body mass index, smoking and alcohol consumption (69). The risk of hypertension (i.e. blood pressure of ≥140/90 mm Hg or taking anti-hypertensive medication) was higher in younger individuals (18–39 years of age) exposed to dioxin-like PCBs (OR=1.34, 95% CI: 1.03–1.74) or DDT (OR=1.42, 95% CI: 1.08–1.85) (64). Interestingly, the risk was lower in older individuals (≥40 years) exposed to non-dioxin-like PCBs or Mirex (64).

PFOS was inversely associated with triacylglycerol and ratio of total cholesterol/high density lipoprotein cholesterol (HDL-C) and positively with HDL-C after controlling for n-3 polyunsaturated fatty acids (70). No association was found between PFOS and low density lipoprotein cholesterol (LDL-C) or non-HDL-C.

### Endocrinology

In one study of 692 middle-aged men and women in Greenland, PCBs and organochlorine pesticides were not associated with impaired glucose tolerance or diabetes (71) (Table IV). Nor were they associated with the surrogate outcomes of fasting glucose, 2-hour glucose, or fasting insulin. Significant inverse associations were found between PCBs and organochlorine pesticides with 2-hour insulin and between PCBs and the homeostasis model assessment of β-cell function.

Dallaire et al. reported on thyroid hormone levels in relation to PCBs, organochlorine pesticides, PBDEs, PFOS and dioxin-like compounds in 623 men and women 18 years of age or older in Nunavik (72). Significant inverse associations were observed for PCBs, hexachlorobenzene, β-hexachlorocyclohexane and PFOS with total triiodothyronine, thyroxine-binding globulin, free thyroxine and/or thyroid-stimulating hormone. p,p′-DDE and PBDEs, however, were not associated with any measure of thyroid function after adjusting for confounders.

### Bone health

Two studies assessed bone strength and bone ultrasound in relation to dioxin-like PCBs or PCBs and organochlorine pesticides, respectively, in peri- and post-menopausal women (73,74) (Table IV). No association was present between bone strength and dioxin-like PCBs (73) or between PCB-153 and bone ultrasound quantitative parameters (74). PCB-156 congener was associated inversely with broadband ultrasound attenuation (indication of bone density and architecture), speed of sound (indication of bone density and elasticity) and stiffness index (indication of rigidity of bone structure).

### Oncology

Breast cancer and exposure to PCBs, organochlorine pesticides, perfluorinated compounds and heavy metals were examined in a case–control study in Greenland (n=31 cases and n=115 controls matched for age and district) (75) (Table IV). Perfluorinated compounds and the sum of persistent organic pollutants were associated positively with breast cancer (OR=1.03, 95% CI: 1.00–1.05 and OR=1.02, 95% CI: 1.01–1.04, respectively). PCB, as a continuous measure, was not associated with breast cancer, but within the highest quartile level, exposure was significantly higher for cases than controls. No association was found with organochlorine pesticides or heavy metals.

DNA hypomethylation, an epigenetic mechanism causing chromosomal instability and alteration of gene expression, was examined in a small study (n=70) consisting mostly of males aged 19–67 years in Greenland (76). PCBs, p,p′-DDE, DDT, other organochlorine pesticides and the sum of persistent organic pollutants were all significantly associated with DNA hypomethylation based on the *Alu* assay but not based on the *LINE-1* assay. The authors suggest that the different mechanisms and transcription patterns in response to cellular stressors accounts for the significant associations observed in one assay and non-significant associations in the other assay.

### Other

The capacities of PCBs and mercury to induce oxidative stress were evaluated in 2 studies of men and women from Nunavik (n=99 each) (77,78) (Table IV). One study examined redox status of coenzyme Q10 and vitamin E (tocopherol) (78) and the other examined oxidation of LDL-C, homocysteine, glutathione peroxidase, glutathione reductase and glutathione (77). Based on tocopherol and coenzyme Q10 redox status, neither PCBs nor mercury increased oxidation, although the elevated ratio of ubiquinone-10 to coenzyme Q10_total_ indicated that the population was experiencing oxidative stress (78). Similarly, in the second study, mercury did not increase oxidation (77). However, PCBs were associated with higher levels of oxidized LDL-C, suggesting that PCBs participate in oxidative stress (77).

## Discussion

A broad range of epidemiological studies have evaluated the possible human health effects of environmental contaminants. However, there are only 60 published studies conducted in Arctic indigenous populations. Several of these studies found association of contaminants with children's immune status, behaviour, neurological function, heart rate variability and thyroid function. In addition, in adults several associations between contaminants and adverse outcomes have been observed, including those on reproductive outcomes, foetal growth and duration of pregnancy, blood pressure and hypertension, thyroid hormones, quantitative measures on bone ultrasound, breast cancer, DNA hypomethylation and oxidative stress. Most of these studies attempted to adjust for several relevant confounders.

Unfortunately, we are still far from being able to make any conclusive statements about the health effects of most environmental contaminants. First, the evidence is largely interspersed with studies showing significant associations and studies showing no associations (i.e. statistical significance not reached). The latter may be due to studies with small sample sizes that are underpowered to detect significant differences. Also, of the studies with larger sample sizes (200+), participation rates ranged from 14 to 95%, with average of 65%, which suggests that there may be issues with the representativeness of study samples. Second, for many contaminant–outcome pairs, we have only 1 or 2 studies contributing data. Conclusions based on observational epidemiological evidence should be based on an accumulation of good quality data that points towards a common direction. Good quality data will come from well-designed observational studies that have representative samples, pre-defined outcomes and subgroup analyses, power calculations and measurement of confounders. Admittedly, epidemiological studies are difficult to conduct in Arctic regions that have small populations and limited accessibility (79). One way to overcome the problems of small sample sizes in individual studies and few studies in Arctic populations is to conduct a meta-analytical synthesis of the literature base on priority contaminants and health outcomes. A necessary component in synthesizing the literature will be to assess the quality of the individual studies. In particular, one issue that came to light during this review is the large number of statistical analyses (i.e. based on different contaminant congeners and subgroups) that were carried out by some studies. Such analyses may result in spuriously significant associations by chance alone. Therefore, it will be important to determine if such subgroup analyses were pre-specified and if they make biological sense.

There are several gaps in the literature, which require future research attention. Published epidemiological studies linking environmental contaminants to health outcomes have been primarily conducted in only 2 regions – Nunavik and Greenland. Additionally, data on obstetrical outcomes are available in indigenous populations of Russian and European North in AMAP reports. It would be informative if data can be accumulated and published from other Arctic populations in Canada (e.g. Inuvialuit Settlement Region, Nunavut, Nunasiavut), Alaska, Russian North and European North. Secondly, while the data in paediatrics and reproductive health are quite extensive, studies available for other areas are notably sparse. For instance, few studies are available on chronic diseases such as cardiovascular disease and diabetes, which make up a large burden of health problems faced by Arctic populations. In addition, only surrogate outcome of cardiovascular disease has been measured. Future studies, therefore, should focus on clinical endpoints as well, such as incidence of heart disease or myocardial infarction. In these respects, analyses of the Adult Inuit Health Survey, which was a cross-sectional survey of about 2,600 Inuit adults from Inuvialuit Settlement Region, Nunavut Territory and Nunatsiavut, and which collected information on heart disease and diabetes, will be useful (80). Lastly, as shown in Figure 3, only a few studies (4 in total) have evaluated PBDEs or perfluorinated compounds. Both have been classified as emerging contaminants in the Arctic (5) and, therefore, more studies on these contaminants and effects on human health are needed.

An important next step is to translate the findings of these and future research studies into meaningful communication messages to policy makers and the populations directly affected by contaminant exposures. Such messages must be evidence-based but also sensitive to the traditions and needs of Arctic regions. Traditional foods are integral to the Arctic peoples’ cultures, through which social cohesion is maintained (5). In addition, traditional foods are a source of nutrients, such as essential fatty acids, which are not adequately obtained from market foods (5). Therefore, messages must incorporate a holistic approach to properly balance the risks of environmental contaminants in traditional foods with their benefits. Such communication is likely to be successful through multistakeholder engagement that includes dialogue from the perspectives of science, policy and communities.

## References

[1] Young TK, Bjerregaard P (2008). Health transitions in Arctic populations.

[2] AMAP (2009). AMAP Assessment 2009: Human Health in the Arctic.

[3] AMAP (2004). Persistent toxic substances, food security and indigenous peoples of the Russian North. Final Report.

[4] AMAP (2014). ArcRisk (Arctic health risks: impacts on health in the Arctic and Europe owing to climate-induced changes in contaminant cycling). Results overview.

[5] Donaldson SG, Van Oostdam J, Tikhonov C, Feeley M, Armstrong B, Ayotte P (2010). Environmental contaminants and human health in the Canadian Arctic. Sci Total Environ.

[6] Laird BD, Goncharov AB, Chan HM (2013). Body burden of metals and persistent organic pollutants among Inuit in the Canadian Arctic. Environ Int.

[7] Young TK (2012). Cardiovascular health among Canada's aboriginal populations: a review. Heart Lung Circ.

[8] Tait H Aboriginal peoples survey, 2006: Inuit health and social conditions. http://www.nativiamericani.it/filevari/89-637-x2008001-eng.pdf.

[9] Bjerregaard P, Young TK, Dewailly E, Ebbesson SOE (2004). Indigenous health in the Arctic: an overview of the circumpolar Inuit population. Scand J Public Health.

[10] Public Health Agency of Canada http://www.phac-aspc.gc.ca/cd-mc/publications/diabetes-diabete/facts-figures-faits-chiffres-2011/pdf/facts-figures-faits-chiffres-eng.pdf.

[11] Grandjean P, Weihe P, Debes F, Choi AL, Budtz-Jørgensen E (2014). Neurotoxicity from prenatal and postnatal exposure to methylmercury. Neurotoxicol Teratol.

[12] Halling J, Petersen MS, Jørgensen N, Jensen TK, Grandjean P, Weihe P (2013). Semen quality and reproductive hormones in Faroese men: a cross-sectional population-based study of 481 men. BMJ Open.

[13] Tang-Peronard JL, Heitmann BL, Andersen HR, Steuerwald U, Grandjean P, Weihe P (2014). Association between prenatal polychlorinated biphenyl exposure and obesity development at ages 5 and 7 y: a prospective cohort study of 656 children from the Faroe Islands. Am J Clin Nutr.

[14] Eggesbo M, Thomsen C, Jorgensen J, Becher G, Odland J, Longnecker MP (2011). Associations between brominated flame retardants in human milk and thyroid-stimulating hormone (TSH) in neonates. Environ Res.

[15] Odland J, Nieboer E, Romanova N, Thomassen Y (2004). Elements in placenta and pregnancy outcome in arctic and subarctic areas. Int J Circumpolar Health.

[16] Odland J, Nieboer E, Romanova N, Thomassen Y, Norseth T, Lund E (1999). Urinary nickel concentrations and selected pregnancy outcomes in delivering women and their newborns among arctic populations of Norway and Russia. J Environ Monit.

[17] Odland J, Nieboer E, Romanova N, Thomassen Y, Lund E (1999). Blood lead and cadmium and birth weight among sub-arctic and arctic populations of Norway and Russia. Acta Obstet Gynecol Scand.

[18] Odland J, Nieboer E, Romanova N, Thomassen Y, Brox J, Lund E (1999). Concentrations of essential trace elements in maternal serum and the effect on birth weight and newborn body mass index in sub-arctic and arctic populations of Norway and Russia. Acta Obstet Gynecol Scand.

[19] Jensen RG, Koch A, Homøe P, Bjerregaard P (2013). Tobacco smoke increases the risk of otitis media among Greenlandic Inuit children while exposure to organochlorines remain insignificant. Environ Int.

[20] Dallaire F, Dewailly É, Vézina C, Muckle G, Weber J-P, Bruneau S (2006). Effect of prenatal exposure to polychlorinated biphenyls on incidence of acute respiratory infections in preschool Inuit children. Environ Health Perspect.

[21] Dallaire F, Dewailly É, Muckle G, Vézina C, Jacobson SW, Jacobson JL (2004). Acute infections and environmental exposure to organochlorines in Inuit infants from Nunavik. Environ Health Perspect.

[22] Dewailly É, Ayotte P, Bruneau S, Gingras S, Belles-Isles M, Roy R (2000). Susceptibility to infections and immune status in Inuit infants exposed to organochlorines. Environ Health Perspect.

[23] Ethier A-A, Muckle G, Bastien C, Dewailly É, Ayotte P, Arfken C (2012). Effects of environmental contaminant exposure on visual brain development: a prospective electrophysiological study in school-aged children. Neurotoxicology.

[24] Boucher O, Burden MJ, Muckle G, Saint-Amour D, Ayotte P, Dewailly É (2012). Response inhibition and error monitoring during a visual go/no-go task in Inuit children exposed to lead, polychlorinated biphenyls, and methylmercury. Environ Health Perspect.

[25] Boucher O, Bastien CH, Saint-Amour D, Dewailly E, Ayotte P, Jacobson JL (2010). Prenatal exposure to methylmercury and PCBs affects distinct stages of information processing: an event-related potential study with Inuit children. Neurotoxicology.

[26] Després C, Beuter A, Richer F, Poitras K, Veilleux A, Ayotte P (2005). Neuromotor functions in Inuit preschool children exposed to Pb, PCBs, and Hg. Neurotoxicol Teratol.

[27] Boucher O, Jacobson SW, Plusquellec P, Dewailly E, Ayotte P, Forget-Dubois N (2012). Prenatal methylmercury, postnatal lead exposure, and evidence of attention deficit/hyperactivity disorder among Inuit children in Arctic Québec. Environ Health Perspect.

[28] Verner M, Plusquellec P, Muckle G, Ayotte P, Dewailly E, Jacobson SW (2010). Alteration of infant attention and activity by polychlorinated biphenyls: unravelling critical windows of susceptibility using physiologically based pharmacokinetic modeling. Neurotoxicology.

[29] Plusquellec P, Muckle G, Dewailly E, Ayotte P, Bégin G, Desrosiers C (2010). The relation of environmental contaminants exposure to behavioral indicators in Inuit preschoolers in Arctic Quebec. Neurotoxicology.

[30] Sandau CD, Ayotte P, Dewailly É, Duffe J, Norstrom RJ (2002). Pentachlorophenol and hydroxylated polychlorinated biphenyl metabolites in umbilical cord plasma of neonates from coastal populations in Quebec. Environ Health Perspect.

[31] Valera B, Muckle G, Poirier P, Jacobson SW, Jacobson JL, Dewailly E (2012). Cardiac autonomic activity and blood pressure among Inuit children exposed to mercury. Neurotoxicology.

[32] Boucher O, Muckle G, Saint-Amour D, Dewailly E, Ayotte P, Jacobson SW (2009). The relation of lead neurotoxicity to the event-related potential P3b component in Inuit children from Arctic Québec. Neurotoxicology.

[33] Saint-Amour D, Roy M-S, Bastien C, Ayotte P, Dewailly E, Després C (2006). Alterations of visual evoked potentials in preschool Inuit children exposed to methylmercury and polychlorinated biphenyls from a marine diet. Neurotoxicology.

[34] Weihe P, Hansen JC, Murata K, Debes F, Jorgensen PJ, Steuerwald U (2002). Neurobehavioral performance of Inuit children with increased prenatal exposure to methylmercury. Int J Circumpolar Health.

[35] Plusquellec P, Muckle G, Dewailly E, Ayotte P, Jacobson SW, Jacobson JL (2007). The relation of low-level prenatal lead exposure to behavioral indicators of attention in Inuit infants in Arctic Quebec. Neurotoxicol Teratol.

[36] Fraser S, Muckle G, Després C (2006). The relationship between lead exposure, motor function and behaviour in Inuit preschool children. Neurotoxicol Teratol.

[37] Krüger T, Long M, Ghisari M, Bonefeld-Jørgensen EC (2012). The combined effect of persistent organic pollutants in the serum POP mixture in Greenlandic Inuit: xenoestrogenic, xenoandrogenic and dioxin-like transactivities. Biomarkers.

[38] Krüger T, Ghisari M, Hjelmborg PS, Deutch B, Bonefeld-Jorgensen EC (2008). Xenohormone transactivities are inversely associated to serum POPs in Inuit. Environ Health.

[39] Long M, Deutch B, Bonefeld-Jorgensen EC (2007). AhR transcriptional activity in serum of Inuits across Greenlandic districts. Environ Health.

[40] Krüger T, Hjelmborg PS, Jönsson BA, Hagmar L, Giwercman A, Manicardi G-C (2007). Xenoandrogenic activity in serum differs across European and Inuit populations. Environ Health Perspect.

[41] Long M, Andersen BS, Lindh CH, Hagmar L, Giwercman A, Manicardi G (2006). Dioxin-like activities in serum across European and Inuit populations. Environ Health.

[42] Bonefeld-Jorgensen EC, Hjelmborg PS, Reinert TS, Andersen BS, Lesovoy V, Lindh CH (2006). Xenoestrogenic activity in blood of European and Inuit populations. Environ Health.

[43] Bonde JP, Toft G, Rylander L, Rignell-Hydbom A, Giwercman A, Spano M (2008). Fertility and markers of male reproductive function in Inuit and European populations spanning large contrasts in blood levels of persistent organochlorines. Environ Health Perspect.

[44] Toft G, Axmon A, Giwercman A, Thulstrup AM, Rignell-Hydbom A, Pedersen HS (2005). Fertility in four regions spanning large contrasts in serum levels of widespread persistent organochlorines: a cross-sectional study. Environ Health.

[45] Stronati A, Manicardi GC, Cecati M, Bordicchia M, Ferrante L, Spanò M (2006). Relationships between sperm DNA fragmentation, sperm apoptotic markers and serum levels of CB-153 and p,p′-DDE in European and Inuit populations. Reproduction.

[46] Spanò M, Toft G, Hagmar L, Eleuteri P, Rescia M, Rignell-Hydbom A (2005). Exposure to PCB and p,p′-DDE in European and Inuit populations: impact on human sperm chromatin integrity. Hum Reprod.

[47] Elzanaty S, Rignell-Hydbom A, Jönsson BA, Pedersen HS, Ludwicki JK, Shevets M (2006). Association between exposure to persistent organohalogen pollutants and epididymal and accessory sex gland function: multicentre study in Inuit and European populations. Reprod Toxicol.

[48] Giwercman A, Rignell-Hydbom A, Toft G, Rylander L, Hagmar L, Lindh C (2006). Reproductive hormone levels in men exposed to persistent organohalogen pollutants: a study of Inuit and three European cohorts. Environ Health Perspect.

[49] Tiido T, Rignell-Hydbom A, Jönsson BA, Giwercman YL, Pedersen HS, Wojtyniak B (2006). Impact of PCB and p,p′-DDE contaminants on human sperm Y:X chromosome ratio: studies in three European populations and the Inuit population in Greenland. Environ Health Perspect.

[50] Krüger T, Spanò M, Long M, Eleuteri P, Rescia M, Hjelmborg PS (2008). Xenobiotic activity in serum and sperm chromatin integrity in European and Inuit populations. Mol Reprod Dev.

[51] Long M, Stronati A, Bizzaro D, Krüger T, Manicardi G-C, Hjelmborg PS (2007). Relation between serum xenobiotic-induced receptor activities and sperm DNA damage and sperm apoptotic markers in European and Inuit populations. Reproduction.

[52] Toft G, Long M, Krüger T, Hjelmborg PS, Bonde JP, Rignell-Hydbom A (2007). Semen quality in relation to xenohormone and dioxin-like serum activity among Inuits and three European populations. Environ Health Perspect.

[53] Mocevic E, Specht IO, Marott JL, Giwercman A, Jonsson BAG, Toft G (2013). Environmental mercury exposure, semen quality and reproductive hormones in Greenlandic Inuit and European men: a cross-sectional study. Asian J Androl.

[54] Kvist L, Giwercman YL, Jönsson BA, Lindh CH, Bonde J-P, Toft G (2012). Serum levels of perfluorinated compounds and sperm Y:X chromosome ratio in two European populations and in Inuit from Greenland. Reprod Toxicol.

[55] Dallaire R, Dewailly É, Ayotte P, Forget-Dubois N, Jacobson SW, Jacobson JL (2013). Exposure to organochlorines and mercury through fish and marine mammal consumption: associations with growth and duration of gestation among Inuit newborns. Environ Int.

[56] Wojtyniak BJ, Rabczenko D, Jönsson BAG, Zvezday V, Pedersen HS, Rylander L (2010). Association of maternal serum concentrations of 2,2′, 4,4′5,5′-hexachlorobiphenyl (CB-153) and 1,1-dichloro-2,2-bis (p-chlorophenyl)-ethylene (p,p′-DDE) levels with birth weight, gestational age and preterm births in Inuit and European populations. Environ Health.

[57] Dallaire R, Muckle G, Dewailly E, Jacobson SW, Jacobson JL, Sandanger TM (2009). Thyroid hormone levels of pregnant Inuit women and their infants exposed to environmental contaminants. Environ Health Perspect.

[58] Toft G, Axmon A, Lindh CH, Giwercman A, Bonde JP (2008). Menstrual cycle characteristics in European and Inuit women exposed to persistent organochlorine pollutants. Hum Reprod.

[59] Lucas M, Dewailly E, Muckle G, Ayotte P, Bruneau S, Gingras S (2004). Gestational age and birth weight in relation to n-3 fatty acids among Inuit (Canada). Lipids.

[60] Muckle G, Dewailly E, Ayotte P, Jacobson SW, Jacobson JL (2004). Contributions of PCBs, pesticides, MeHg and n-3 fatty acids to fetal growth and motor development in Inuit infants in Arctic Quebec. Neurotoxicology.

[61] Pereg D, Dewailly E, Poirier GG, Ayotte P (2002). Environmental exposure to polychlorinated biphenyls and placental CYP1A1 activity in Inuit women from northern Québec. Environ Health Perspect.

[62] Lagueux J, Pereg D, Ayotte P, Dewailly E, Poirier GG (1999). Cytochrome P450 CYP1A1 enzyme activity and DNA adducts in placenta of women environmentally exposed to organochlorines. Environ Res Sect A.

[63] Foldspang A, Hansen JC (1990). Dietary intake of methylmercury as a correlate of gestational length and birth weight among newborns in Greenland. Am J Epidemiol.

[64] Valera B, Jørgensen ME, Jeppesen C, Bjerregaard P (2013). Exposure to persistent organic pollutants and risk of hypertension among Inuit from Greenland. Environ Res.

[65] Valera B, Dewailly E, Poirier P (2013). Association between methylmercury and cardiovascular risk factors in a native population of Quebec (Canada): a retrospective evaluation. Environ Res.

[66] Nielsen ABS, Davidsen M, Bjerregaard P (2012). The association between blood pressure and whole blood methylmercury in a cross-sectional study among Inuit in Greenland. Environ Health.

[67] Valera B, Dewailly E, Poirier P (2009). Environmental mercury exposure and blood pressure among Nunavik Inuit adults. Hypertension.

[68] Valera B, Dewailly E, Poirier P (2008). Cardiac autonomic activity and blood pressure among Nunavik Inuit adults exposed to environmental mercury: a cross-sectional study. Environ Health.

[69] Luoma PV, Näyhä S, Pyy L, Hassi J (1995). Association of blood cadmium to the area of residence and hypertensive disease in Arctic Finland. Sci Total Environ.

[70] Château-Degat M-L, Pereg D, Dallaire R, Ayotte P, Dery S, Dewailly E (2010). Effects of perfluorooctanesulfonate exposure on plasma lipid levels in the Inuit population of Nunavik (Northern Quebec). Environ Res.

[71] Jørgensen ME, Borch-Johnsen K, Bjerregaard P (2008). A cross-sectional study of the association between persistent organic pollutants and glucose intolerance among Greenland Inuit. Diabetologia.

[72] Dallaire R, Dewailly É, Pereg D, Dery S, Ayotte P (2009). Thyroid function and plasma concentrations of polyhalogenated compounds in Inuit adults. Environ Health Perspect.

[73] Paunescu A-C, Ayotte P, Dewailly É, Dodin S (2013). Dioxin-like compounds are not associated with bone strength measured by ultrasonography in Inuit women from Nunavik (Canada): results of a cross-sectional study. Int J Circumpolar Health.

[74] Côté S, Ayotte P, Dodin S, Blanchet C, Mulvad G, Petersen HS (2006). Plasma organochlorine concentrations and bone ultrasound measurements: a cross-sectional study in peri-and postmenopausal Inuit women from Greenland. Environ Health.

[75] Bonefeld-Jorgensen EC, Long M, Bossi R, Ayotte P, Asmund G, Krüger T (2011). Perfluorinated compounds are related to breast cancer risk in Greenlandic Inuit: a case control study. Environ Health.

[76] Rusiecki JA, Baccarelli A, Bollati V, Tarantini L, Moore LE, Bonefeld-Jorgensen EC (2008). Global DNA hypomethylation is associated with high serum-persistent organic pollutants in Greenlandic Inuit. Environ Health Perspect.

[77] Bélanger M-C, Dewailly E, Berthiaume L, Noël M, Bergeron J, Mirault M-E (2006). Dietary contaminants and oxidative stress in Inuit of Nunavik. Metabolism.

[78] Bélanger M-C, Mirault M-E, Dewailly E, Berthiaume L, Julien P (2008). Environmental contaminants and redox status of coenzyme Q10 and vitamin E in Inuit from Nunavik. Metabolism.

[79] Odland JØ, Nieboer E (2012). Human biomonitoring in the Arctic. Special challenges in a sparsely populated area. Int J Hyg Environ Health.

[80] Saudny H, Leggee D, Egeland G (2012). Design and methods of the Adult Inuit Health Survey 2007–2008. Int J Circumpolar Health.

